# What is the incubation period for listeriosis?

**DOI:** 10.1186/1471-2334-13-11

**Published:** 2013-01-10

**Authors:** Véronique Goulet, Lisa A King, Véronique Vaillant, Henriette de Valk

**Affiliations:** 1 Infectious Diseases Department, Institut de Veille Sanitaire, 12 rue du val d'osne, 94415, Saint Maurice, France

**Keywords:** Listeriosis, Incubation, *Listeria monocytogenes*, Outbreak

## Abstract

**Background:**

Listeriosis is a foodborne infection with a low incidence but a high case fatality rate. Unlike common foodborne diseases, the incubation period can be long. The first incubation periods were documented during a large listeriosis outbreak published in 1987 by Linnan and al. in the New England Journal of Medicine (range: 3 days to 70 days). Data on the incubation period of listeriosis are scarce. Our study aim was to estimate precisely the incubation period of listeriosis using available data since 1987.

**Methods:**

We estimated the incubation period of listeriosis using available published data and data from outbreak investigations carried out by the French National Institute for Public Health Surveillance. We selected cases with an incubation period calculated when a patient had a single exposure to a confirmed food source contaminated by *Listeria monocytogenes*.

**Results:**

We identified 37 cases of invasive listeriosis (10 cases with central nervous system involvement (CNS cases), 15 bacteraemia cases and 12 pregnancy-associated cases) and 9 outbreaks with gastroenteritis. The overall median incubation period of invasive listeriosis was 8 days (range: 1–67 days) and differed significantly by clinical form of the disease (p<0.0001). A longer incubation period was observed for pregnancy-associated cases (median: 27.5 days; range: 17–67 days) than for CNS cases (median: 9 days; range: 1–14 days) and for bacteraemia cases (median: 2 days; range: 1–12 days). For gastroenteritis cases, the median incubation period was 24 hours with variation from 6 to 240 hours.

**Conclusions:**

This information has implications for the investigation of food borne listeriosis outbreaks as the incubation period is used to determine the time period for which a food history is collected. We believe that, for listeriosis outbreaks, adapting the exposure window for documenting patients’ food histories in accordance with the clinical form of infection will facilitate the identification of food products as the source of contamination. We therefore propose to take an exposure window of 14 days before the diagnosis for CNS and bacteraemia cases, and of 6 weeks before the diagnosis, for pregnancy-associated cases.

## Background

Listeriosis is an uncommon bacterial infection that is potentially fatal in the foetus, in newborns and immunocompromised adults. *Listeria monocytogenes* (Lm) cause invasive listeriosis with central nervous system involvement (meningitis, meningoencephalitis) and bactaeremia with a high case fatality rate (20% to 30%) [1,2]. Lm also cause non-invasive disease such as gastroenteritis [3]. Lm is widely distributed in the environment and can contaminate a wide variety of foods [4,5]. Lm has the ability to grow at low temperatures (+4°C) and is destroyed by heat. The epidemiology of listeriosis has been partly elucidated since the 1980’s, when foodborne transmission was established [6,7]. Vehicles of transmission of Lm are mostly ready-to-eat foods [5].

In infected hosts, Lm crosses the intestinal epithelium barrier via transcytosis and invades the mesenteric lymph nodes and the blood [8]. The majority of bacteria become trapped in the liver and therefore are rapidly cleared form the circulatory system. Surviving bacteria replicate in hepatocytes. Early recruitment of polymorphonuclear cells leads to hepatocyte lysis, and thereby bacterial release. If the infection is not controlled at this stage, for instance because of severe immunodepression, a secondary bacteraemia develops. Bacteria circulating in the blood, either free or associated with leucocytes are disseminated to sanctuary sites by transgressing the blood-brain barrier or the placental barrier [9]. In pregnant hosts the bacteria can probably cross the endothelium of the maternal blood-vessels, followed by the entry into the fetal circulatory system of the placental villi [10].

In foodborne diseases the incubation period is the delay between the consumption of a contaminated product and the onset of first symptoms of the disease. Unlike common food borne diseases such as salmonellosis, the incubation period of listeriosis can be long. Multiple aspects of listeriosis make the determination of a precise incubation period difficult. Firstly, Lm can contaminate a large variety of foods, which hinders the identification of an infected food source. Secondly, as the incubation period is thought to be variable and long, products consumed during a long period (usually 30 days) are suspected. Thirdly, many products contaminated by Lm are products that can be kept during several days or weeks and can therefore be eaten by the patient on multiple occasions. Therefore data on incubation period referring to single exposures are scarce. The first data on the incubation period for listeriosis were published in 1987 about a Californian outbreak associated with cheese that involved more than 100 cases [6]. Linnan reported already the difficulty in identifying sufficient cases with a single exposure from which an incubation period could be calculated. He subsequently identified a median incubation period of 31 days (range, 11 to 70) in four patients with single exposure.

Since this outbreak, authors of scientific publications and text books for epidemiologists and clinicians refer to this incubation period [11,12]. In 2006, in a paper on a nationwide outbreak associated with frankfurters involving 108 cases in the United States of America, Mead mentioned that in this outbreak “the average incubation period for invasive listeriosis is shorter than generally assumed” but could not document it more accurately as many patients had recurrent exposures to the implicated product [13].

Our study aimed to estimate precisely the incubation period of listeriosis using available published data and data obtained during outbreak investigations carried out by the Institut de Veille Sanitaire (French Institute for Public Health Surveillance, InVS). Our goal is to enhance the efficiency of investigations targeted towards identifying foods at the origin of listeriosis outbreaks by documenting as precisely as possible the incubation period, for the different forms of invasive listeriosis.

## Methods

We searched in PubMed, papers published between January, 1980, and January, 2012 (inclusive), using the terms ((Disease outbreaks OR cross-infection OR Clusters) AND (listeriosis OR listeria *monocytogenes*)) AND (Food OR investigation).

By reviewing the 288 retrieved records, we identified 42 reports on outbreaks or clusters of listeriosis, and among them 16 reports documenting food borne point-source listeriosis outbreaks. Additional documented food-borne listeriosis cases or clusters were identified by reviewing the reference lists of the retrieved reports (n=3). Furthermore, we reviewed french listeriosis investigation reports of the Institut de Veille Sanitaire and identified data on incubation period in three reports of unpublished outbreaks and one report of a sporadic foodborne case.

A precise incubation period was defined as the delay between the date of consumption of a contaminated food and the date of onset of clinical symptoms or, if not available, of the date of the first Lm positive biological sample taken from the patient.

We reviewed the selected 23 reports to identify cases with a documented incubation period, calculated when a patient had a single exposure to a confirmed food source of contamination. The estimated incubation period of cases with multiple consumptions or with no precise date of consumption (i.e. date of purchase of the contaminated product) were classified as “approximate incubation period”.

Cases of invasive listeriosis were classified as:

– cases with central nervous system involvement (CNS cases): Lm isolated from cerebrospinal fluid (CSF), or from blood in a patient with clinical symptoms of CNS involvement

– bacteraemia cases: Lm isolated from blood cultures with no clinical symptoms of CNS involvement

– pregnancy-associated cases: Lm isolated in blood from a pregnant woman, or in samples from placenta, a foetus, a stillbirth, or a newborn less than one month of age.

Cases of listeria gastroenteritis were defined as:

– cases with gastrointestinal symptoms and Lm isolates recovered from stool.

– or cases with gastrointestinal symptoms epidemiologically linked to cases of listeriosis confirmed by Lm isolation.

Results are expressed as a median [range] for continuous variables. Associations between quantitative and qualitative variables were assessed using the Kruskal-Wallis test. Statistical analysis and box plots were performed using Stata8^®^. For each clinical form of invasive listeriosis, we calculated the median incubation period and the first and the third quartile (in days). For gastroenteritis cases, we report for each outbreak, the number of cases, the median and the range of the incubation period (in hours).

## Results and discussion

**Figure 1:**
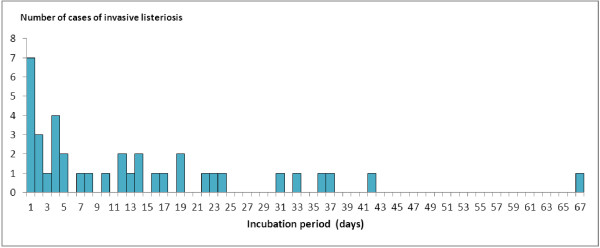
Distribution of the incubation period (in days) for 37 invasive cases of listeriosis.

Among the 23 reports, we identified 15 reports with precise documented incubation periods for invasive listeriosis (Table 1). In total, a precise incubation period was documented for 37 invasive cases (10 CNS cases, 15 bacteraemia cases and 12 pregnancy-associated cases). For invasive listeriosis, the overall median incubation period was 8 days (range: 1–67 days) (Figure 1).

**Table 1:** Estimates of incubation period of 37 cases with documented single exposure, by clinical form of invasive listeriosis

**Clinical form**	**Country**	**Year**	**Ref**	**Implicated food**	**Number of cases**	**Incubation period (days)**
**CNS involvement**						
	UK	1988	[14]	cheese	1	1
	France	1993	[15]	rillettes (pork pâté)	2	10*,12*
	France	1995	[16]	brie (cheese)	1	8*
	France	1999	[17]	rillettes (pork pâté)	1	14*
	Belgium	2001	[18]	frozen cake	1	4
	France	2003	†	spreadable sausage	1	2
	Austria	2008	[19]	jellied pork tongue	1	14
	Germany	2009	[20]	cheese	1	13
	Norway	2010	[21]	camembert (cheese)	1	4
**Bacteraemia**						
	Finland	1989	[22]	salted mushrooms	1	1
	Italy	1993	[23]	rice salad	2	1,1
	USA	1998	[13]	frankfurter	1	2
	Austria	2008	[19]	jellied pork tongue	1	2*
	Norway	2010	[21]	camembert (cheese)	9	3,7,1,1,5,1,12,4,5
	France	2011	†	horse minced meat	1	4
**Pregnancy associated**						
	USA	1989	[24]	shrimp	2	19,23
	France	1993	[15]	rillettes (pork pâté)	1	42*
	France	1995	[16]	brie (cheese)	6	17*,22*,31*,33*,37*,67*
	France	1997	†	pont l’evêque (cheese)	2	36*,24*
	France	2000	†	mozzarella	1	19*

* Duration between the consumption of contaminated food and the date of the biological sampling of the patient.

† French Institute of Public Health Surveillance, InVS (unpublished data).

**Figure 2:**
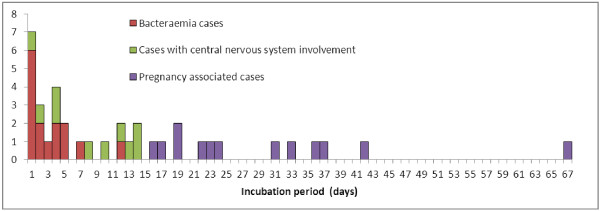
Distribution of the incubation period (in days) of 37 invasive cases of listeriosis by clinical form of disease.

**Figure 3:**
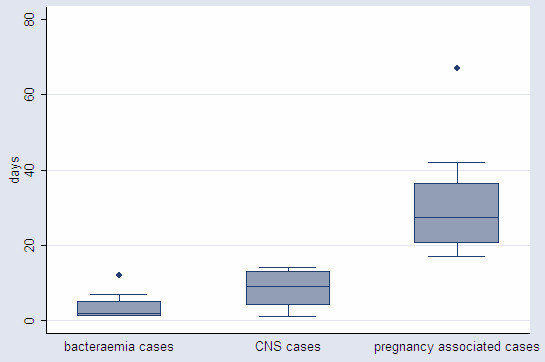
**Distribution of incubation period for each clinical form of 37 invasive cases of listeriosis (box-plot). **Line in the middle of boxes represents median of data. Boxes extend from the 25th percentile (X_[25]_) to 75th percentile (X_[75]_), representing interquartile range (IQR). Lines emerging from boxes extend to upper and lower adjacent values. The upper adjacent value is defined as the largest data point ≤X_[75] _+ 1.5 x IQR. The lower adjacent value is defined as the smallest data point ≥X_[25] _– 1.5 x IQR. Dots are outliers (every point more than 1.5 x IQR from the end of a box).

The incubation period differed significantly by clinical form of invasive listeriosis (Kruskall-wallis, p<0.0001) (Figure 2). A longer incubation period was observed for pregnancy-associated cases (median: 27.5 days; range: 17–67 days; 1^st^ and 3^rd^ quartiles: 20, 36 days) than for CNS cases (median: 9 days; range: 1–14 days; 1^st^ and 3^rd^ quartiles: 4,13 days) and for bacteraemia cases (median: 2 days; range: 1–12 days; 1^st^ and 3^rd^ quartiles: 1,5 days) (Figure 3).

Estimates of an approximate incubation period were available for 14 cases (2 CNS cases, 6 bacteraemia cases, 6 pregnancy-associated cases) (Table 2).

**Table 2:** Source of information for estimates of incubation period of 16 cases with imprecise exposure by clinical form of invasive listeriosis

**Clinical form**	**Country**	**Ref**	**Implicated food**	**Year**	**Number of cases**	**Available Information**	**Maximum Incubation period**
**CNS involvement**							
	France	[16]	brie (cheese)	1993	1	date of purchase	19
	Denmark	[25]	meat dish	2010	1	date of production	21
**Bacteraemia**							
	Belgium	[26]	camembert	1994	1	consumed a few days ago	5
	Norway	[21]	camembert	2007	3	2 possible dates of exposure	3-15, 2-6, 19-21
	Denmark	[25]	meat dish	2009	2	date of production	21,27
	UK	[27]	sandwich	2011	3	2 possible dates of exposure	1-4 to 8-13
**Pregnancy associated**							
	France	[15]	rillettes (pork pâté)	1993	5	date of purchase	16,18,28,38,88

For gastroenteritis, 9 outbreaks were reviewed. For each outbreak, the median and range of incubation periods are shown in Table 3. For gastroenteritis cases, the median incubation period was 24 hours. Incubation periods varied from 6 to 240 hours.

**Table 3:** Source of information for incubation period for cases with listeria gastroenteritis

**Country**	**Year**	**Ref**	**Implicated food**	**Number of cases**	**Incubation period**	
					**Median**	**Range**
Italy	1993	[23]	rice salad	14	18h	6-36h
USA	1994	[28]	chocolate milk	45	20h	9-32h
Italy	1997	[29]	corn salad	1566	24h	6-51h
New-Zealand	2000	[30]	corn beef	31	24h	12-168h
Finland	1999	[31]	smoked trout	5		<27h
USA, LA	2001	[32]	turkey	16	25h	6-49h
Sweden	2001	[33]	cheese	17	31h	10-240
Japan	2001	[34]	cheese	28	36h	<24->144
Austria	2008	[19]	jellied pork tongue	13	24h	24-48h

The results of this study clearly demonstrate that the listeriosis incubation period is shorter than generally assumed and varies according to the clinical form of the disease. Not surprisingly the shortest incubation period is observed for listeria associated gastroenteritis (one day) with an incubation period quite similar in duration to other enteric bacteria such as *Salmonella*. The incubation period is also short for bacteraemia cases, with a median of 2 days and is longer for CNS cases, with a median of 9 days (p<0.05). The range of incubation periods for CNS cases was, however, wide (1 to 14 days) compared with bacteraemia cases (1-7 days) if we exclude one outlier bacteraemia case with a much longer incubation of 12 days (Figure 2). With a median of 27.5 days and a range of 17-67 days, pregnancy-associated forms have a much longer incubation period than other clinical forms. A likely explanation is that there is a delay between bacteraemia and infection of the foetus due to the time necessary for Lm to colonize the placenta and to induce a placentitis that is at the origin of the fetal infection. An experimental study on pregnant guinea pig supports the hypothesis that the placenta is relatively protected from infection [35]. Once colonized, the placenta acts as a nidus of infection for the mother resulting in massive reseeding of maternal organs, where Lm cannot be cleared until trafficking is interrupted by expulsion of the infected placental tissue. This hypothesis is consistent with the delay observed between ingestion of a contaminated food and foetal infection.

Situations in which an incubation period can be precisely documented are exceptional for listeriosis. Evidence of the link between a contaminated food and illness can be obtained in outbreak situations, but such evidence is rarely available for sporadic cases. The situation best adapted to documentation of a precise incubation period is an outbreak linked to a single meal, since the moment of consumption of the incriminated product coincides with the moment of contamination. When the outbreak is due to a product consumed over a longer period of time or on a regular basis, it is extremely difficult to identify the exact date of contamination. To be accurate, only single exposures should be used for the calculation of the incubation period. Our study of all documented incubation periods with a point source exposure during a 32 year period was able to identify 37 precise incubation periods. It is the most complete and comprehensive series of such cases analyzed to address this question.

Approximate incubation periods that we calculated without precise point-source contamination are consistent with the precise estimates (Table 2). One outbreak reported in this table suggests that the incubation period of invasive listeriosis may be longer than our estimates [25]. All the eight patients of this outbreak had a comorbidity impairing their immunity and received ready-made food delivered at home by the same catering company. As the only meal in common was prepared on April 14, 2009, the authors suggested that this meal was at the origin of the outbreak. If this was the case, the range of incubation periods for the 7 bacteraemia cases would be 21–27 days and 21 days for the only CNS case. We were reluctant to take this outbreak into account for the calculation of precise incubation periods since most of these patients had daily delivery of meals from this catering company. As Lm can easily colonize kitchen surfaces, different meals prepared on subsequent days may also have been contaminated by cross contamination or by a contaminated ingredient used in the preparation of several meals.

The wide range of incubation periods observed for each form may be related to varying levels of contamination of food, the quantity of contaminated food consumed, the virulence of the Lm strain or the immunological status of the patient. In our study, the majority of bacteraemia cases with documented incubation periods were observed during hospital outbreaks among persons with comorbidities that impair immunity. When Lm was identified by blood culture, they all received an antibiotic treatment that effectively reduced colonisation of the CNS system. Interestingly in an outbreak that occurred after a single meal in Austria, one 72 year old attendee of the meal fell ill with fever and diarrhoea, recovering within two days, but then developed CNS symptoms on day 14 [19]. Diagnosis of listeriosis with CNS involvement was confirmed by Lm isolation in CSF. In this outbreak, another attendee hospitalized two days after the meal for fever and diarrhea, had blood cultures positive for Lm. He was subsequently treated and did not develop CNS symptoms. These observations suggest that CNS involvement occurs after transient bacteraemia and thus has a longer incubation period. In France, 75% of the bacteraemia forms of listeriosis are diagnosed in patients with comorbidity. Febrile patients with comorbidity are more likely to have blood drawn for culture and to be diagnosed in the case of bacteraemia. In contrast, blood cultures are uncommon for febrile persons without comorbidity. If these unrecognized bacteraemia are not treated, Lm can subsequently infect the CNS. The French surveillance data show that 69% of listeriosis cases in patients without comorbidity have CNS involvement.

The exposure windows considered when interviewing patients about their food consumption is a delicate issue. By taking a wide exposure window, one gains in terms of sensitivity by including a variety of foods consumed that are more or less food habits of the consumer. By taking a smaller exposure window, one reduces recall biases and gains in specificity by limiting the number of foods consumed. In a case-control study of sporadic *Salmonella Enteritidis* infections, Molbak compared food exposure data obtained for an exposure window corresponding with the maximum incubation period (7 days) to food exposure data for an exposure window corresponding to the most relevant incubation period (1 day) [36]. The conclusion was that for common food exposures, exposure classification that corresponds to the most common period of incubation rather than the maximum period is more accurate. Our study suggests that, to be efficient, food interviews for listeriosis outbreak investigations should use different exposure windows according to the clinical form of the disease. We suggest that *Listeria* gastroenteritis cases should be interviewed about their exposures during the two days before their first symptoms and pregnancy-associated cases, 6 weeks prior to their first symptoms. For other forms, the most appropriate option would be to interview bacteraemia cases about the 7 days prior to symptom onset and CNS cases during 14 days prior to their first symptoms. However, in the context of routine surveillance, it is sometimes difficult to discriminate bacteraemia patients with mental confusion from cases with CNS involvement. Therefore it could be more pragmatic to use a unique exposure window of 14 days when interviewing these patients for surveillance purpose.

Another important outcome of a more precisely documented listeriosis incubation period relates to prevention messages issued by health authorities when a contaminated product is withdrawn from the market. In France, persons who consumed Lm contaminated products are advised to watch carefully for any symptoms of listeriosis during a period of 2 months after exposure. Based on our results, we recommend that these messages should be adapted to recommend 6 weeks of vigilance for pregnant women and 2 weeks for other exposed individuals.

## Conclusions

Our results indicate that the incubation period for listeriosis varies according to the clinical presentation of the disease. A much longer incubation period was observed for pregnancy-associated cases than for cases with other clinical forms. This information has implications for the investigation of food borne listeriosis outbreaks as the incubation period is used to determine the time period for which a food history is collected. We propose for CNS and bactaeriemia cases to take an exposure window of 14 days before the diagnosis and for pregnancy-associated cases, 6 weeks before the diagnosis. We believe that, for listeriosis outbreaks, adapting the exposure windows used to document patients’ food histories according to the clinical form of infection will facilitate the identification of food products as the source of contamination.

## Competing interests

No competing interests. This work was supported by the Institut de Veille Sanitaire which is funded by the French Ministry of health.

## Authors’ contributions

VG is at the origin of this work, conducted the search in Pubmed and analysed all the papers selected, and drafted the manuscript. VV, LK and HV contributed by documenting incubation periods during listeriosis outbreak investigations and critically revised the manuscript. They have approved the final manuscript.

## Pre-publication history

The pre-publication history for this paper can be accessed here:

http://www.biomedcentral.com/1471-2334/13/11/prepub
